# Immunologic constant of rejection as a predictive biomarker of immune checkpoint inhibitors efficacy in non-small cell lung cancer

**DOI:** 10.1186/s12967-023-04463-2

**Published:** 2023-09-19

**Authors:** Alice Mogenet, Pascal Finetti, Emilie Denicolai, Laurent Greillier, Pascaline Boudou-Rouquette, François Goldwasser, Gwenael Lumet, Michele Ceccarelli, Daniel Birnbaum, Davide Bedognetti, Emilie Mamessier, Fabrice Barlesi, François Bertucci, Pascale Tomasini

**Affiliations:** 1 Multidisciplinary Oncology and Therapeutic Innovations Department, Aix Marseille Univ, APHM, INSERM, CNRS, CRCM, Hôpital Nord, Marseille, France; 2 Centre de Recherche en Cancérologie de Marseille (CRCM), INSERM UMR1068, CNRS UMR725, Laboratoire d’Oncologie Prédictive, Aix Marseille Univ, Marseille, France; 3 Department of Medical Oncology, Cochin Hospital, AP-HP, Paris, France–University of Paris Descartes, ARIANE, CARPEM, Paris, France; 4 Sylvester Comprehensive Cancer Center, Department of Public Health Sciences, University of Miami Miller School of Medicine, Miami, FL USA; 5 Division of Translational Medicine, Research Branch, Sidra Medicine, Doha, Qatar; 6 Department of Internal Medicine and Medical Specialties, University of Genoa, Genoa, Italy; 7 Paris-Saclay University and Medical Oncology, Gustave Roussy, Cancer Campus, Villejuif, France; 8 Department of Medical Oncology, Institut Paoli-Calmettes, Aix Marseille Univ, 232, Bd de Sainte-Marguerite, 13009 Marseille, France

**Keywords:** Lung cancer, ICR signature, Immune therapy, Biomarkers, Immune checkpoints inhibitors, Transcriptomics

## Abstract

**Background:**

Anti-PD1/PDL1 immune checkpoint inhibitors (ICI) transformed the prognosis of patients with advanced non-small cell lung cancer (NSCLC). However, the response rate remains disappointing and toxicity may be life-threatening, making urgent identification of biomarkers predictive for efficacy. Immunologic Constant of Rejection signature (ICR) is a 20-gene expression signature of cytotoxic immune response with prognostic value in some solid cancers. Our objective was to assess its predictive value for benefit from anti-PD1/PDL1 in patients with advanced NSCLC.

**Methods:**

We retrospectively profiled 44 primary tumors derived from NSCLC patients treated with ICI as single-agent in at least the second-line metastatic setting. Transcriptomic analysis was performed using the nCounter^®^ analysis system and the PanCancer Immune Profiling Panel. We then pooled our data with clinico-biological data from four public gene expression data sets, leading to a total of 162 NSCLC patients treated with single-agent anti-PD1/PDL1. ICR was applied to all samples and correlation was searched between ICR classes and the Durable Clinical Benefit (DCB), defined as stable disease or objective response according to RECIST 1.1 for a minimum of 6 months after the start of ICI.

**Results:**

The DCB rate was 29%; 22% of samples were classified as ICR1, 30% ICR2, 22% ICR3, and 26% ICR4. These classes were not associated with the clinico-pathological variables, but showed enrichment from ICR1 to ICR4 in quantitative/qualitative markers of immune response. ICR2-4 class was associated with a 5.65-fold DCB rate when compared with ICR1 class. In multivariate analysis, ICR classification remained associated with DCB, independently from PDL1 expression and other predictive immune signatures. By contrast, it was not associated with disease-free survival in 556 NSCLC TCGA patients untreated with ICI.

**Conclusion:**

The 20-gene ICR signature was independently associated with benefit from anti-PD1/PDL1 ICI in patients with advanced NSCLC. Validation in larger retrospective and prospective series is warranted.

**Supplementary Information:**

The online version contains supplementary material available at 10.1186/s12967-023-04463-2.

## Background

Despite significant improvement in systemic treatments during the last few years, advanced non-small-cell lung cancer (NSCLC) remains the leading cause of cancer-related death worldwide [1]. Recent advances in lung cancer biology knowledge led to molecular dismemberment of the disease, leading to the development of targeted therapies. This changed the lung cancer prognosis thanks to personalized therapeutic approach [2]. Beyond oncogenic addictions, cancer immunology breakthrough allowed for the emergence of immune checkpoint inhibitors (ICI) [3]. These drugs, which target the PD1-PDL1 axis, are now widely used in the first- and second-line settings for advanced NSCLC [4–6]. Unfortunately and in contrast with targeted therapies, only 20% of patients respond to ICI as single agent and up to 50% when combined with chemotherapy [4–6]. This suggests an incomplete knowledge of the molecular determinants of immune responsiveness. Furthermore, life-threatening or fatal immune-related adverse events occur in some patients. Consequently, there is still a long way to achieve immune dissection of targeted immunotherapies-mediated effects, and an urgent need to identify biomarkers able to predict anti-PD1/PDL1 ICI efficacy [7].

To date, evaluation of PDL1 expression on tumors cells by immunohistochemistry (IHC) using the PD-L1 IHC 22C3 pharmDx assay (CPS > 50%) is the only approved companion diagnostics for pembrolizumab, whereas the PD-L1 IHC 22C3 pharmDx and Ventana PD-L1 (SP142) have status as complementary diagnostics for nivolumab and atezolizumab. It is however an imperfect predictive biomarker which is not sufficient to face the most important existing challenge in the field of immunotherapy [8]. The main reason given is the operator’s variability to evaluate PDL1 protein expression and its spatial heterogeneity [9]. Furthermore, the use of different IHC tests with the different anti-PD1 and anti-PD-L1 ICI adds to these difficulties. Several other predictive biomarkers have been studied, but none of them appears to be reliable and reproducible enough to improve on the predictive significance of PDL1 expression, despite a better consideration of various biological features underlying ICI efficacy. Tumor mutational burden (TMB), thought to reflect the amount of neo-antigens on tumor cells by quantifying non-synonymous mutations in coding areas, failed to show a clear survival difference when used alone and remains difficult to standardize for routine use [10, 11]. In a same way, quantification of tumor-infiltrating lymphocytes and other circulating biomarkers appeared to be interesting prognostic biomarkers, but not predictive of ICI efficacy [12, 13]. In addition, interferon gamma (IFN-Ɣ) pathway is known to be involved in PDL1 expression and is a key feature to several anti-tumor immune expression signatures, such as the T cell-inflamed signature (TIS) [14]. The independent predictive value of this signature was validated in a prospective series of 37 NSCLC treated with nivolumab, independently from PDL1 expression by IHC [15], and more recently in patients treated with pembrolizumab, in a pan-cancer clinical trial performed across 20 tumor types, including lung cancer [16]. Finally, methylation profiles and microbiota assessment turned out to be hardly operable in daily care [17–19].

Thus, determinants of ICI response in NSCLC appeared to be many and various, depending on tumor and microenvironment features. Considering the relative failure of these descriptive biomarkers, more functional biomarkers such as transcriptomic signatures could become key parameters to solve this complex equation. Besides the TIS signature, other signatures including IFN-Ɣ-related genes showed also positive predictive value with atezolizumab in 115 NSCLC in the POPLAR study [20] and with durvalumab in 97 NSCLC [21]. Gene expression signatures (GES) associated with enrichment in tertiary lymphoid structures (TLS) have also been generated in other cancers, including the Coppola’s 12-chemokine signature [22]. Other prognostic and/or predictive immune signatures were reported in series ranging from 21 to 67 patients with NSCLC [23–26]. The ICR (Immunologic Constant of Rejection) signature is defined as an immune phenotype quantifying the expression of 20 genes, all involved in anti-tumor immunity. Selected genes are reflecting main anti-tumor immune pathways, such as Th1 signaling (*IFNG, TBX21, CD8A/B, IL12B, STAT1* and *IRF1*), Th1 chemoattraction (*CXCL9, CXCL10* and *CCL5*) and cytotoxic functions (*GNLY, PRF1, GZMA, GZMB* and *GZMH*). Interestingly, the expression of these pro-cytotoxic transcripts is associated with inhibition of suppressive mechanisms, known as ICIs (*CD274, PDCD1, IDO1, CTLA4* and *FOXP3*) [27].

In early-stage breast cancer, we showed that ICR signature divided the tumors in four ICR groups reflecting a clinically and biologically relevant immune continuum, and displayed independent predictive values for metastasis-free survival (MFS) and for achievement of pathological complete response (pCR) to neo-adjuvant chemotherapy [28, 29]. ICR4 tumors strongly expressed ICR signature and showed longer MFS and higher pCR rate to chemotherapy than other ICR tumors. Similarly, we recently showed the independent prognostic value of ICR in soft tissue sarcomas [30]. Regarding ICI, a potential positive predictive value for response was reported in melanoma [31].

Given these correlations in other cancers, its immune relevance, and the growing place of ICIs in advanced NSCLC treatment, we assessed the ICR signature as a potential predictive biomarker for ICI response in a cohort of 162 patients with advanced NSCLC treated with anti-PD1/PDL1 ICI. Our secondary objectives were to compare the predictive value of ICR with that of other immune signatures and to assess its prognostic value in NSCLC untreated with ICI.

## Methods

### Patients’ populations and gene expression profiling

Our own series included formalin-fixed paraffin-embedded (FFPE) tumor samples from the previously biopsied primary tumor of 77 consecutive patients with advanced NSCLC who had received an anti-PD1/PDL1 ICI as single agent outside clinical trials in our institution (Hôpital Nord, Marseille, France). All clinical data were collected from the patients’ electronic medical record. This non-interventional retrospective study was approved by our institutional review board under the number 2019_93. All patients had given their signed written informed consent for the use of archived material for research purpose. Tumor RNA was extracted using “Maxwell^®^ RSC Instrument” (Promega) shortly after microtome dissection to prevent nucleic acid degradation. After extraction, RNA quantification and quality control were done using the Nano-Drop ND-1000 technology (ThermoFisher). Forty-four out of 77 tumor samples fulfilled the quality and quantity thresholds for downstream transcriptomic analysis. This latter was performed using nCounter® technology Dx analysis as recommended by Nanostring, based on microscopic imaging counting relative abundance of 770 transcripts present in the “PanCancer Immune Profiling Panel”, which included the 20 ICR genes. Briefly, the profiling required three successive steps: a first hybridization step to capture target sequences, followed by a purification step, and finally a quantification step using the Digital Analyzer processor.

In order to expand our series, we collected gene expression and clinico-pathological data from four publicly available data sets [15, 23, 32, 33] of NSCLC patients treated with a single-agent anti-PD1/PDL1 ICI and with available clinical outcome. In these published data sets, gene expression profiling had been done using Nanostring technology or RNA-Sequencing. The final pooled data set included 162 metastatic NSCLC patients clinically annotated, notably in term of clinical benefit after ICI treatment (Additional file 2: Table S1). Finally, in order to assess the eventual prognostic value of ICR outside any ICI treatment, we collected The Cancer Genome Atlas (TCGA) lung adenocarcinoma (LUAD) data set [34] and the TCGA lung squamous cell carcinoma (LUSC) data set [35] including 515 and 502 patients respectively with gene expression data.

### Gene expression data analysis

Nanostring data processing and normalization were performed using the nSolver^™^ 4.0 analysis software. Briefly, data processing of raw counts was done with background subtraction defined by the geometric mean of the eight negative control probes. Next, normalization was done with the geometric mean algorithm using the 40 housekeeping and the six positive control probes. Processed data were then log_2_-transformed. Before analysis of pooled data sets, several steps of data processing were applied. The first step was the normalization of each set separately. It was done in R using Bioconductor and associated packages; we used quantile normalization for the available processed log_2_-transformed data. We then applied to each data set separately several multigene signatures.

First, the ICR classifier based on consensus clustering (CC) analysis of the expression levels of 20 representative immune genes (namely *CCL5, CD274, CD8A, CD8B, CTLA4, CXCL9, CXCL10, FOXP3*, *GNLY, GZMA, GZMB, GZMH, IDO1, IFNG, IL12B, IRF1, PDCD1, PRF1, STAT1,* and *TBX21*) as previously described [29]. Briefly, the CC analysis was performed in R using the Bioconductor package “ConsensusClusterPlus” [36] setting as input parameters 5000 repetitions, 80% item resampling (pItem), a number of groups (k) fixed to 4 (in order to have all datasets stratified with the same number of classes, 4 being the optimal number of groups for the TCGA cohort [29], and the use of an agglomerative hierarchical clustering with ward criterion (Ward.D2) inner and complete outer linkage. Second, we applied several other transcriptional signatures related to immune response: metagenes of signatures of 28 innate and adaptative immune cell subpopulations defined by Bindea et al. [37], activation score of IFN-α, IFN-Ɣ, and TNF-α pathways [38], cytolytic activity score [39], and antigen processing and presentation machinery score (APMS) [40]. We also applied the TP53 activation score [38], and three potential predictors of response to ICI: PDL1 (*CD274*) expression, T cell-inflamed signature (TIS) [14], and tertiary lymphoid structures (TLS) signature [22]. These three predictors were tested as binary variables using the first quintile as cut-off, thus defining the “low” (first quintile) and “high” (four last quintiles) classes. Third, *PDL1* gene expression levels (*CD274*) were extracted from each data set and were standardized within each data set using the NSCLC population as a reference to be comparable across data sets and to exclude bias from population heterogeneity. Of note, we verified the correlation between mRNA and protein expressions of PDL1 in lung cancer samples (cell lines and clinical tumors; Additional file 1: Fig S1).

### Statistical analysis

The continuous variables were described by median and range, and the binary variables by numbers and percentage. Correlations between tumor classes and clinico-pathological or molecular variables were analyzed using the one-way analysis of variance (ANOVA) or the Fisher’s exact test when appropriate. Our primary endpoint was the Durable Clinical Benefit (DCB), a relevant criterion of ICI efficacy considering the challenge and the expected paradigm shift to overcome natural course of the disease and induce prolonged response [41]. We defined DCB as stable disease or objective response according to RECIST 1.1 for a minimum of 6 months after the start of ICI. This definition has been mainly previously published among ICI literature and especially biomarkers literature [42–44]. Uni- and multivariate analyses for DCB were done using logistic regression (glm; significance estimated by specifying a binomial family for models with a logit link). The variables submitted to univariate analysis included patients’ age (continuous value), sex (male *vs* female), smoker status (current *vs* former *vs* non-smoker), pathological type (squamous *vs* non-squamous), mutational status (mutated *vs* non-mutated), and classifications based on ICR (ICR2-4 *vs* ICR1), TIS (“high” *vs* “low”), TLS (“high” *vs* “low”) and PDL1 expression (“high” *vs* “low”). Variables with a *p*-value < 0.05 in univariate analyses were tested in multivariate analyses. In this meta-analysis of five independent sets, we used the test of homogeneity in a fixed-effects model to provide evidence about whether the effect sizes are measuring a common effect size. The prognostic analysis in the TCGA set used the Disease-Free Survival (DFS) as endpoint, calculated from the date of diagnosis until the date of distant relapse. Follow-up was measured from the date of diagnosis to the date of last news for event-free patients. Survivals were calculated using the Kaplan–Meier method and curves were compared with the log-rank test. All statistical tests were two-sided at the 5% level of significance. Statistical analysis was done using the survival package (version 2.43-3) in the R software (version 3.5.1; http://www.cran.r-project.org/). We followed the reporting REcommendations for tumor MARKer prognostic studies (REMARK criteria) [45].

## Results

### Patients’ population and ICR classification

**Fig. 1:**
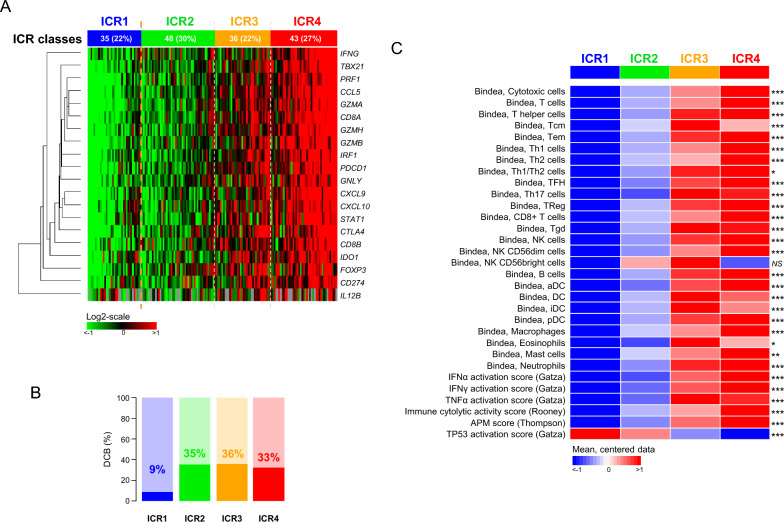
ICR classification of 162 NSCLC samples treated with anti-PD1/PDL1 ICI and correlations with immune variables. **A** Expression heatmap of the 20 ICR genes in 162 tumor samples. The samples (columns) are ordered from left to right according to their increasing ICR score. The 20 genes (raws) are ordered from top to bottom according to hierarchical clustering with uncentered Pearson correlation distance and centroid agglomerative method as parameters. The expression levels are color-coded according to the indicated color scale. Above the heatmap, the four ICR classes are indicated. **B** Correlation of ICR classes with DCB. The percentage of patients with DCB is indicated for each class. **C** Heatmap representation of expression scores of several immune-related variables and non-immune related variables in the four ICR classes. The mean scores are shown as median-centered according to the colored scale shown at the bottom. The p-values of comparison between the four classes (one-way ANOVA test) are shown on the right (*NS* not significant; * < 0.05; **, < 0.01; ***, < 0,001)

We profiled our series of 44 tumor samples that we coupled with 118 profiled samples from four published public datasets [15, 23, 32, 33], obtaining a cohort of 162 advanced NSCLC patients treated with anti-PD1/PDL1 ICI. Baseline patients’ characteristics are summarized in Table 1. They were representative of patients treated by daily care: the median patients’ age was 60 years, 67% of informative patients were male, 89% were active or former smokers, 65% of tumors were non-squamous pathological type, and 38% displayed somatic mutations (*KRAS*, then *EGFR*, then *NRAS*, *STK11*, and *ROS1*). Regarding our primary endpoint, 47 patients (29%) experienced a DCB after ICI treatment and 115 (71%) did not. ICR classification of the 162 tumors defined 35 tumors (22%) as ICR1, 48 (30%) as ICR2, 36 (22%) as ICR3, and 43 (26%) as ICR4 (Fig. 1A).

**Table 1:** Clinico-pathological characteristics of patients and samples

Characteristics		N
Patient’s age, years	Median (range)	60 (26–86)
Sex	Female	41 (33%)
	Male	84 (67%)
Smoker status	Current smoker	46 (46%)
	Former smoker	43 (43%)
	Non-smoker	11 (11%)
Pathological type	Non-squamous	22 (38%)
	Squamous	47 (35%)
Mutational status	Mutated^a^	22 (38%)
	Wild-type	36 (62%)

^a^2 *EGFR*, 17 *KRAS*, 1 *NRAS*, 1 *ROS1*, 1 *STK11* mutations

### ICR classification and clinico-pathological and immune variables

We first searched for correlations between the four ICR classes and different variables. There was no significant correlation with the following clinico-pathological features: patients’ age and sex, smoker status, pathological type, and mutational status (Additional file 2: Table S2). By contrast, a significant correlation existed with the achievement of DCB (p = 2.67E-02, Fisher’s exact test), with DCB rates equal to 9% in ICR1 *versus* 35% in ICR2, 36% in ICR3, and 33% in ICR4 (Fig. 1B).

We also found strong correlations with immunity-related features with a continuum between the four classes (from ICR1 to ICR4) for nearly all features (Fig. 1C; Additional file 2: Table S2). All but one Bindea’s signatures for immune cell subsets [37] showed a strong enrichment from ICR1 to ICR4, notably cytotoxic cells, T-cells, CD8 + T-cells, Th1 cells, TFH cells and activated NK CD56^dim^ cells (p < 1.00E-15). Among T-helper cells, the Th1/Th2 ratio increased from ICR1 to ICR4. This increasing anti-tumor activation was associated with increasing immune cell subsets involved in antigen presentation, such as activated dendritic cells (aDC), DC, B-cells, and macrophages. Such enrichment in cell subsets was confirmed using more functional immune signatures, with gradual enrichment from ICR1 to ICR4 for activation scores of IFN-α, IFN-Ɣ, and TNF-α pathways (p < 1.00E-08), for the cytolytic activity score [39] (p < 1.00E-30) and for the APMS score [40] (p < 1.00E-15). Conversely, the TP53 pathway activation score [38] decreased from ICR1 to ICR4 (p < 1.00E-05).

### ICR classification and DCB after ICI treatment

**Fig. 2:**
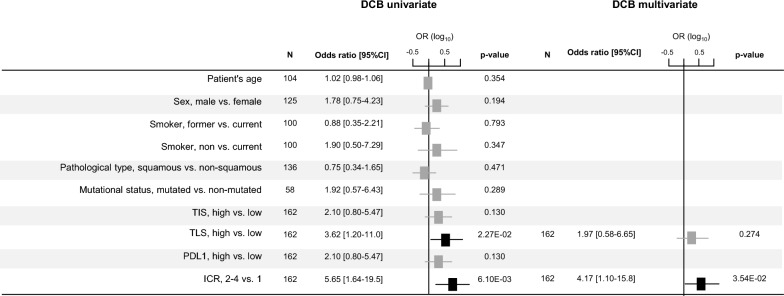
Uni- and multivariate analyses for DCB. Forest plots of univariate (**A**) and multivariate (**B**) analyses for DCB after ICI. The Odds Ratios are log_10_-transformed

Based on the absence of difference in the DCB rate between the ICR2, 3 and 4 classes (p = 0.937, Fisher’s exact test), we pooled them into the ICR2-4 class that we then compared to the ICR1 class. The DCB rates were 9% in ICR1 (3/35 patients) *versus* 35% in ICR2-4 (44/127 patients), corresponding to a 5.65 Odds Ratio (OR) (95% CI 1.64–19.51; p = 6.10E-03, logit function) in ICR2-4 *versus* ICR1 in univariate analysis (Fig. 2). Interestingly, analysis of homogeneity in a fixed-effects model revealed homogeneity between the five data sets in term of correlation between DCB rate and ICR1/2–4 classes (p = 0.950; Additional file 1: Fig S2). Notably, in our own cohort of 44 patients, the DCB rates were 11% in ICR1 (1/9 patients) *versus* 40% in ICR2-4 (10/35 patients), corresponding to a 3.20 OR (95% CI 0.35–29.01). None of the clinico-pathological variables tested in univariate analysis displayed significant correlation with the achievement of DCB (Fig. 2): patients’ age and sex, smoker status, pathological type, and mutational status.

Then, we compared this predictive value of ICR for DCB to that of PDL1 expression and two immune signatures (TIS, TLS) previously reported as predictive for response to ICI. In univariate analysis (Fig. 2), the four variables showed a positive association with DCB with an OR for DCB superior to 2 in the “TIS-high”, “TLS-high”, “PDL1-high”, and ICR2-4 patients when compared with the “TIS-low”, “TLS-low”, “PDL1-low”, and ICR1 patients respectively. However, likely due to the number of patients, the correlation was not significant for TIS and PDL1. However, it was significant for TLS (p = 2.27E-02, logit function) and ICR (p = 6.10E-03, logit function) signatures. In multivariate analysis (Fig. 2), only ICR remained significant, suggesting stronger and independent predictive value.

### ICR classification and correlation with survival without ICI treatment

**Fig. 3:**
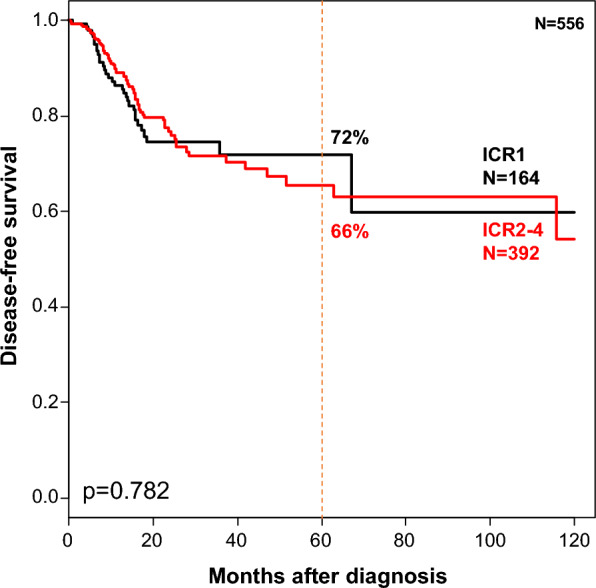
Disease-free survival according to the ICR classification in NSCLC untreated with ICI. Kaplan–Meier DFS curves in early-stage patients with lung adenocarcinoma and squamous cell carcinoma (TCGA dataset) according to the two ICR classes (ICR1 *versus* ICR2-4). The p-value indicated is for the log-rank test

We assessed the eventual prognostic value of ICR in an early-stage NSCLC population (TCGA dataset of 556 M0 patients including 408 with lung adenocarcinoma and 148 with lung squamous carcinoma) naive from ICI and with available post-operative disease-free survival (DFS). A total of 164 samples were classified as ICR1 (29%), 127 as ICR2 (23%), 188 as ICR3 (34%), and 77 as ICR4 (14%), with similar correlations with immune variables as those described in our population treated with ICI (Additional file 2: Table S3). With a median follow-up of 158 months, 101 (18%) displayed a DFS event and the 5 year DFS was 67% (95% CI 61–74). It was 72% (95% CI 63–82) in the ICR1 class, and 66% (95% CI 58–74) in the ICR2-4 class, suggesting no prognostic value of ICR classification (p = 0.782, log-rank test; Fig. 3).

## Discussion

Immune checkpoint inhibitors are considered as a revolution for patients with advanced NSCLC and became a therapeutic standard for NSCLC without oncogenic addiction in European and US Guidelines regardless of PDL1 expression [46, 47]. Nevertheless, extended efficacy with acceptable safety profile remains too scarce and identification of predictive biomarkers for ICI efficacy became a major challenge. Here, we showed the independent predictive value of the ICR signature for efficacy of single-agent anti-PD1/PDL1 ICI in the largest retrospective multicentric clinical reported cohort of patients with NSCLC.

Our approach tested ICR in an independent series of samples, thus avoiding the problem of overfitting. A total of 162 pre-therapeutic cancer samples informative for DCB after ICI and large-scale gene expression profile were available, allowing not only to test our hypothesis in uni- and multivariate analyses, but also to test many other gene signatures and modules relevant to immune response. Our series was profiled using the Nanostring® technology, already available in clinical routine for breast cancer prognostication (CE-IVD label) and more adapted for daily care, notably for small FFPE or frozen biopsies as often available in NSCLC. The DCB was chosen to explore ICR predictive value because of its clinical relevance and consistency in the immunotherapy field [43, 48].

As described in other cancers such as breast cancer [28], sarcomas [30], or colon cancer [49], we found an immunological continuum in NSCLC from ICR1 to ICR4 classes. There was an increasing enrichment in scores reflecting the amount of different immune cell types, notably T-cells, cytotoxic T-cells, Th1-cells, CD8 + T-cells, and antigen-presenting cells, and functional scores reflecting IFN-Ɣ pathway activation, cytolytic activity, and antigen presentation machinery. Conversely, the activation score of TP53 pathway decreased from ICR1 to ICR4, in agreement with the higher rate of inactivating *TP53* mutations reported in ICR4 previously reported in breast cancer [29].

A strong association between ICR classes and DCB was observed: the DCB rates were 9% in ICR1 patients *versus* 35% in ICR2-4 patients, corresponding to a 5.65 OR. Importantly, there was homogeneity through the five pooled data sets in term of correlation between DCB rate and ICR classes. This predictive value strengthens the potential clinical role of ICR signature and the correlation between tumor immunogenicity and ICI efficacy. Interestingly, ICR was more predictive than other potential biomarkers such as PDL1 expression, TIS and TLS signatures, whereas, as expected, no tested clinico-pathological feature showed any predictive value. Although associated with the same immune and biological variables in the TCGA set than in our 162 patients, the ICR signature was not associated with survival in TCGA patients untreated with ICI, suggesting that ICR is not broadly prognostic in NSCLC and might be only informative in the presence of an ICI treatment. However, since the lymphocyte infiltration seems to have some prognostic role in NSCLC [50], this absence of prognostic value of ICR in our TCGA analysis may appear surprising and clearly deserves further comparative assessment of both biomarkers in the same clinical series.

All 20 genes of ICR signature are involved in tumor immune pathways and previously published data highlighted their functional implication and clinical relevance. Despite the failure of therapeutic application, IFN-Ɣ remains the most described cytokine involved in anti-tumor immune response. On one hand, IFN-Ɣ upregulation at the early phase of immune response is obviously a major step of anticancer immunity using various downstream mechanisms. On the other hand, IFN-Ɣ is also known for its regulatory role in a further step of immune response, also able to enhance tumor growth and PDL1 expression [51]. Moreover, *TBX21*, known as a transcription factor of IFN-Ɣ, already showed a negative association with NSCLC prognosis by mediating tumor growth [52]. IL12 is also involved in triggering IFN-Ɣ to switch CD4 helper differentiation into Th1 cells and was already described across expression signatures as a prognostic biomarker in NSCLC [53]. Downstream IFN-Ɣ pathway is inducing STAT1 activation achieving various biological functions such as IRF1 expression and tumor growth regulation, but STAT1 activation is also described for enhancing tumor progression through chronic inflammation responsible for controversial therapeutic implication [54]. ICR signature also includes genes involved in Th1 chemo-attraction through IFN-Ɣ pathway, like *CXCL9* and *CXCL10*, regulating naïve T cells migration, differentiation and activation and inhibiting angiogenesis [55]. Other genes included in ICR signature are mainly involved in direct cytotoxic function, such as *CD8*, *GNLY*, *GZMA*, *GZMB* and *GZMH*. Finally, immunosuppressive mechanisms known as checkpoint inhibitors are considered in the signature and widely involved in ICI efficacy such as *CD274*, *PDCD1* or *CTLA4* but also *IDO1* and *FOXP3*. *IDO1* is involved in tryptophan metabolism and known to enhance the PI3K/AKT signaling and also with the T regulatory cell generation in tumor microenvironment, thus associated with poor prognosis in various tumor types [56, 57]. ICR provides a surrogate for the ability of immune response to attack the cancer cells if the checkpoint is inhibited.

Our result is consistent with other studies that reported a predictive value of immune signatures for the benefit of ICI in lung cancer [7]. These signatures, ranging from 4 to 59 genes, include genes coding for proteins involved in tumor antigenicity and T cell priming/activation, trafficking of T cells and infiltration into tumors, recognition of cancer cells by T cells, infiltration by inhibitory cells, and immune checkpoints receptors or ligands. Many of them include IFN-Ɣ-related genes, such as the 18-gene TIS associated with objective response and PFS after pembrolizumab in the KEYNOTE-028 trial [16], the 8-gene “T-effector and IFN-Ɣ signature” associated with overall survival (OS) after atezolizumab in 115 NSCLC patients in the POPLAR study [20], or the 4-gene “IFN-Ɣ signature” associated with objective response and PFS after durvalumab in 97 NSCLC patients [21]. Other predictive immune signatures were reported in smaller NSCLC series ranging from 21 to 67 patients [23–26]. Interestingly, the ICR signature remained the sole variable significant in our multivariate analysis for DCB prediction, when confronted to the TIS and TLS signatures. The predictive value of ICR for the response to systemic anti-cancer treatments has already been reported in retrospective studies. In early-stage breast cancer, we showed that ICR signature was independently associated with achievement of pathological complete response (pCR) to neo-adjuvant chemotherapy [28, 29]. Regarding ICI, a potential positive predictive value of ICR was reported for clinical response in melanoma [31], and more recently for the pathological response to neo-adjuvant chemotherapy combined with pembrolizumab in breast cancer [58]. Such predictive value should not be limited to these two cancers, and clearly further studies are warranted in other indications.

Our study displays strengths and limitations. The first limitations are related to the retrospective nature and its associated biases such as heterogeneity and missing data. For example, our population included patients treated by ICI as single agent in different metastatic lines, whereas a wide majority of patients now receive ICI in the first-line setting; the samples used for the transcriptomic analyses were obtained at various stages of the disease (early and late) leading to some heterogeneity in our population considering temporal tumor heterogeneity; relevant data were missing such as the TMB or PDL1 expression by IHC, even if we replaced it by mRNA expression that correlates with protein expression in lung cancers (Additional file 1: Fig S1). Second, we used the DCB as efficacy endpoint, rather than overall survival (OS) and progression free survival (PFS), but DCB has now been widely used among cancer immunotherapy literature, reinforcing its clinical significance. Finally, the number of samples analyzed was relatively small when compared to the frequency of NSCLC, but to our knowledge, our series is the largest series of gene expression profiling of NSCLC patients treated with ICI. The strength of our results lies in: (i) the number of 162 samples that, to our knowledge, makes our series the largest gene expression study reported so far in this setting; (ii) its originality, being the first one to describe the predictive value of ICR signature for response to ICI in NSCLC; (iii) a population of patients treated in the community setting, likely more reflective of real-life than a selected patient population within a clinical trial; (iv) the biological relevance of ICR, its predictive value in the context of ICI treatment and absence of prognostic value in absence of ICI; and (v) the small number of genes included (20 genes), which should facilitate its clinical application once validated.

## Conclusion

We showed the predictive value of ICR in a large, composite, and multicentric cohort. Even if a validation is required in larger retrospective cohorts and in prospective trials to confirm the robustness of our results in the first-line setting and chemotherapy combination, ICR displays a promising signal of efficacy in the so far disappointing immune biomarkers field. In a near future, predicting ICI efficacy will be a major challenge with three pivotal questions: (i) when? Balanced with the new data on ICI use in early-stage NSCLC and the re-challenge in widely pretreated patients [59–61]; (ii) how? By assessing various techniques to develop composite predictors likely including clinical, biological, genomic, transcriptomic, proteomics, and spatial data to better consider the different drivers of immune response; and (iii) what? Considering the amount of new potential therapeutic immune targets, starting by the 20 genes of the signature, and the wide possibilities of treatment associations and sequences.

### Supplementary Information

**Additional file 1: Figure S1.** Correlation of PDL1 mRNA (CD274) and protein expression in lung carcinoma samples. A/ Correlation in the 329 cancer cell lines (grey) and in the 66 lung cancer cell lines (blue) in the DepMap database. B/ Correlation in the 234 lung adenocarcinoma clinical samples in the TCGA series. **Figure S2.** Analysis of homogeneity in a fixed-effects model revealed homogeneity between the five data sets in term of correlation between DCB rate and ICR1/2-4 classes (p=0.950).

**Additional file 2: Table S1.** List of NSCLC data sets analyzed. **Table S2.** ICR classification and correlation with clinico-pathological and biological variables in the 162 NSCLC samples. **Table S3.** ICR classification and correlations with biological variables in the 556 TCGA NSCLC samples. **Table S4.** Normalized gene expression data for the 44 NSCLC samples included in the study.

## Data Availability

The datasets supporting the conclusions of this article are included within the article and its additional files (Additional files 1, 2).

## Abbreviations

ANOVA: One-way analysis of variance
APMS: Antigen processing and presentation machinery score
CC: Consensus clustering
CRCM: *Centre de Recherche en Cancérologie de Marseille*
DCB: Durable clinical benefit
DFS: Disease free survival
FFPE: Formalin-fixed paraffin-embedded
GES: Gene expression signatures
ICI: Immune checkpoint inhibitors
ICR: Immunologic constant of rejection
IFN-Ɣ: Interferon Gamma
IHC: Immunohistochemistry
LUAD: Lung adenocarcinoma
LUSC: Lung squamous cell carcinoma
MFS: Metastasis-free survival
NSCLC: Non-small cell lung cancer
OR: Odds Ratio
OS: Overall survival
pCR: Pathological complete response
PD1: Programmed cell death protein 1
PDL1: Programmed death ligand 1
PFS: Progression-free survival
REMARK: REcommendations for tumor MARKer prognostic studies
TCGA: The cancer genome atlas
TMB: Tumor mutational burden
TIS: T cell-inflamed signature
TLS: Tertiary lymphoid structures
