# Supplementary material for: Immunologic constant of rejection as a predictive biomarker of immune checkpoint inhibitors efficacy in non-small cell lung cancer
Source: J Transl Med. 2023 Sep 19;21:637. doi: 10.1186/s12967-023-04463-2 (PMC10507965; doi:10.1186/s12967-023-04463-2)
Supplement: Supplementary file 1 — Additional file 1: Figure S1. Correlation of PDL1 mRNA (CD274) and protein expression in lung carcinoma samples. A/ Correlation in the 329 cancer cell lines (grey) and in the 66 lung cancer cell lines (blue) in the DepMap database. B/ Correlation in the 234 lung adenocarcinoma clinical samples in the TCGA series. Figure S2. Analysis of homogeneity in a fixed-effects model revealed homogeneity between the five data sets in term of correlation between DCB rate and ICR1/2-4 classes (p=0.950). [file 12967_2023_4463_MOESM1_ESM.pptx]

## Slide 1
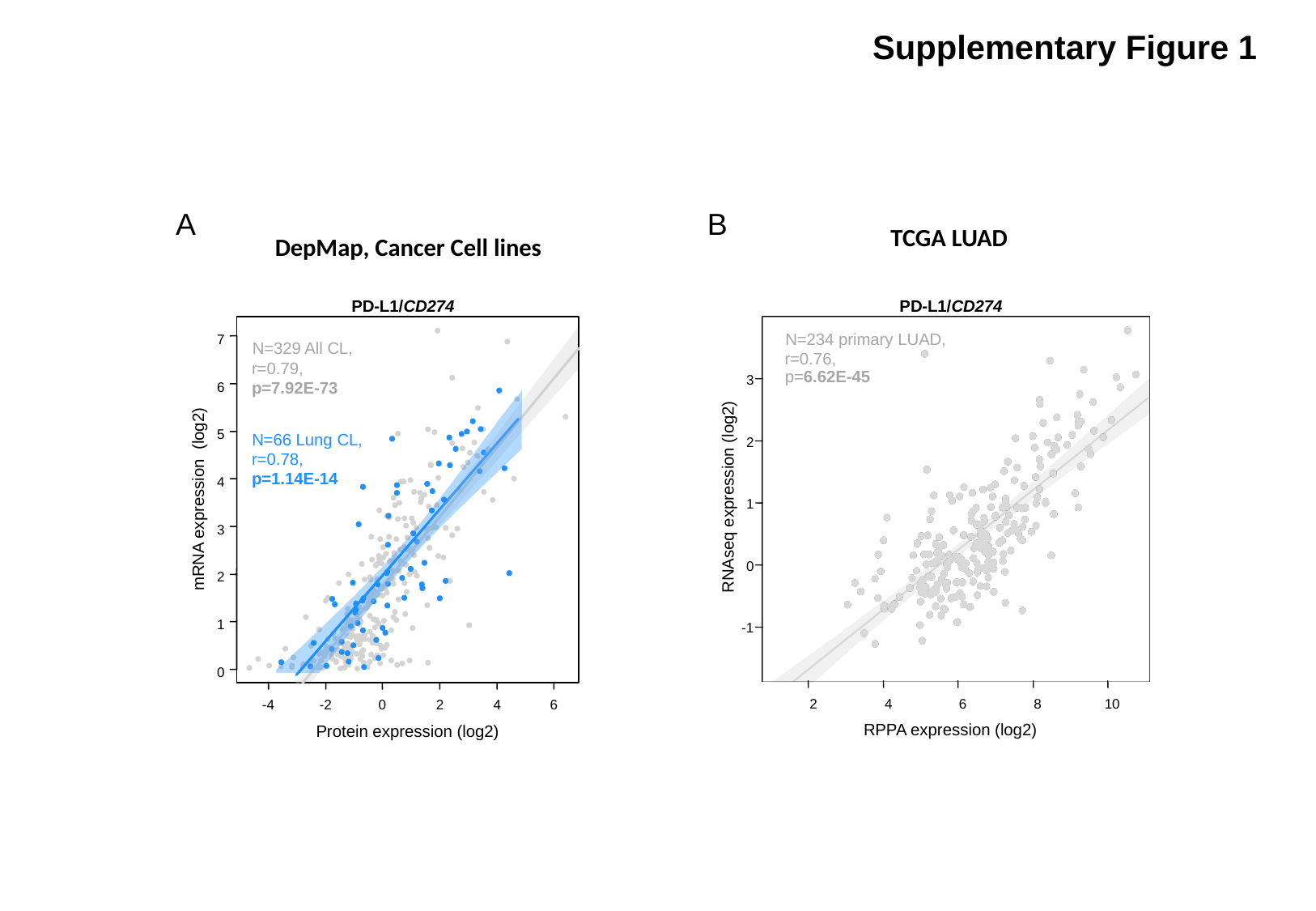

Supplementary Figure 1
A
B
TCGA LUAD
DepMap, Cancer Cell lines
PD-L1/CD274
7
6
5
4
mRNA expression (log2)
3
2
1
0
-4
-2
0
2
4
6
Protein expression (log2)
N=329 All CL,
r=0.79,
p=7.92E-73
N=66 Lung CL,
r=0.78,
p=1.14E-14
PD-L1/CD274
N=234 primary LUAD,
r=0.76,
p=6.62E-45
3
2
RNAseq expression (log2)
1
0
-1
2
4
6
8
10
RPPA expression (log2)

## Slide 2
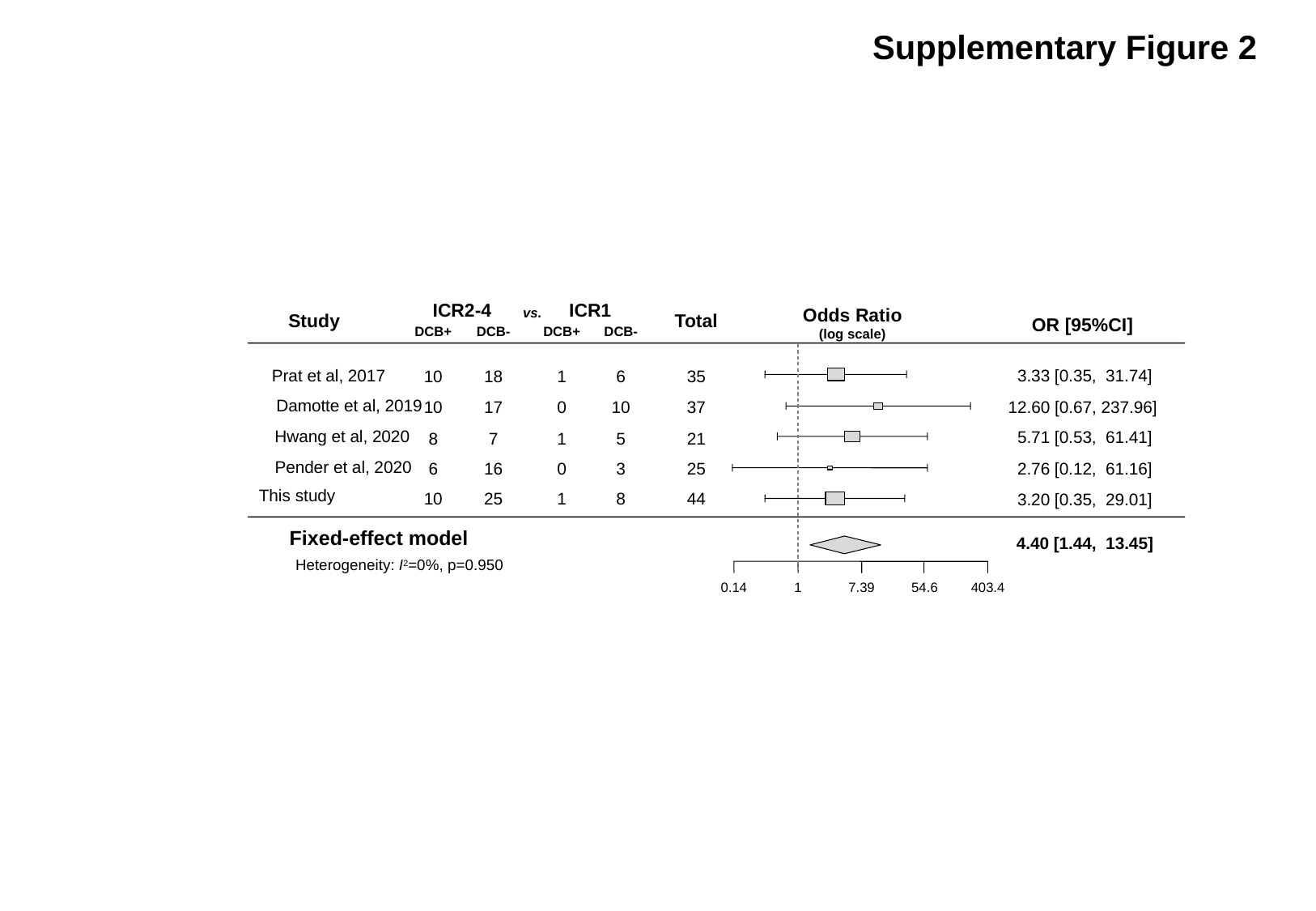

Supplementary Figure 2
ICR2-4
ICR1
vs.
Total
Study
DCB+
DCB-
DCB+
DCB-
Prat et al, 2017
10
18
1
6
35
Damotte et al, 2019
10
17
0
10
37
Hwang et al, 2020
8
7
1
5
21
Pender et al, 2020
6
16
0
3
25
10
25
1
8
44
Fixed-effect model
Heterogeneity: I2=0%, p=0.950
Odds Ratio
(log scale)
OR [95%CI]
0.14
1
7.39
54.6
403.4
 3.33 [0.35, 31.74]
12.60 [0.67, 237.96]
 5.71 [0.53, 61.41]
 2.76 [0.12, 61.16]
 3.20 [0.35, 29.01]
 4.40 [1.44, 13.45]
This study
